# Biosensing with Förster Resonance Energy Transfer Coupling between Fluorophores and Nanocarbon Allotropes

**DOI:** 10.3390/s150614766

**Published:** 2015-06-23

**Authors:** Shaowei Ding, Allison A. Cargill, Suprem R. Das, Igor L. Medintz, Jonathan C. Claussen

**Affiliations:** 1 Department of Mechanical Engineering, Iowa State University, 2104 Black Engineering, Ames, IA 50011, USA; E-Mails: swding@iastate.edu (S.D.); acargill@iastate.edu (A.A.C.); srdas@iastate.edu (S.R.D.); 2 Center for Bio/Molecular Science & Engineering Code 6900, US Naval Research Laboratory, Washington, DC 20375, USA; E-Mail: igor.medintz@nrl.navy.mil

**Keywords:** Förster Resonance Energy Transfer (FRET), graphene, carbon nanotubes, carbon dots, carbon nanoparticles, biosensor

## Abstract

Nanocarbon allotropes (NCAs), including zero-dimensional carbon dots (CDs), one-dimensional carbon nanotubes (CNTs) and two-dimensional graphene, exhibit exceptional material properties, such as unique electrical/thermal conductivity, biocompatibility and high quenching efficiency, that make them well suited for both electrical/electrochemical and optical sensors/biosensors alike. In particular, these material properties have been exploited to significantly enhance the transduction of biorecognition events in fluorescence-based biosensing involving Förster resonant energy transfer (FRET). This review analyzes current advances in sensors and biosensors that utilize graphene, CNTs or CDs as the platform in optical sensors and biosensors. Widely utilized synthesis/fabrication techniques, intrinsic material properties and current research examples of such nanocarbon, FRET-based sensors/biosensors are illustrated. The future outlook and challenges for the research field are also detailed.

## 1. Introduction

In the post-silicon technology era, there has been a surge of research focused on several functional nanomaterials; no doubt, the allotropes of carbon are among the most sought-after candidates under consideration due to the unique combination of physicochemical properties they offer. Over the last several years, graphene, carbon nanotubes (CNTs) and carbon dots (CDs)/nanoparticles have revolutionized electrical, optical, thermal, mechanical, chemical and sensing phenomena [1,2,3,4,5]. Their unique physiochemical properties, which include exceedingly high room temperature carrier mobility [6], surface area-to-volume ratios and, hence, high surface reactivity [6], quenching efficiency [7], and mechanical strength and flexibility [8], are potentially advantageous for a wide variety of biological applications including biosensing [2,3,9,10,11], drug delivery [12], therapeutics [13,14] and catalysis [15,16]. In particular, their nanoscale size and dimensionality (CDs: zero-dimensional; CNTs: one-dimensional; and graphene: two-dimensional), as well as biocompatibility make them well suited for interfacing with biological components. Furthermore, their inherent high quenching efficiencies are of particular importance for fluorescent-based biosensors that utilize the absence or presence of Förster resonant energy transfer (FRET) to monitor molecular dynamics, such as protein conformational changes, protein-protein interactions and protein-DNA interactions for biosensing [9,17], intracellular imaging [18,19], and biomolecular logic [20,21].

FRET is the transfer of energy from one excited molecular fluorophore (the donor molecule) to another fluorophore (the acceptor molecule) via intermolecular dipole-dipole interactions [22,23]. The FRET efficiency (*E*) between two fluorophores is inversely proportional (1/R^6^) to the distance (*R*) between the donor and acceptor; FRET generally occurs with donor-to-acceptor distances between 1 and 8 nm [24]. This short distance dependency enables FRET to be used as a tool to determine nanometer-length distances between dye-quencher conjugates [25]. Therefore, FRET-based sensors have the ability to transduce a near-field (nanoscale), non-measureable interaction into a far-field measureable signal, an attribute that exceeds the capability of optical microscopy and biologically-destructive high-resolution microscopy techniques, such as scanning electron microscopy or tunneling electron microscopy [22,26].

This review will focus on discussing the most current body of research regarding sensors and biosensors comprising graphene, CNTs or CDs that are functionalized to use FRET as a means to transduce or amplify chemical/biological recognition events. The review will illustrate widely used synthesis/fabrication techniques, highlight key intrinsic material properties and portray current research examples of nanocarbon allotropes (NCAs) utilized in FRET-based sensing and biosensing. The review concludes with a summary of the outlook of the field and recommendations for future work. While many reviews related to FRET-based sensing have been published, this review focuses specifically on FRET coupling between fluorophores and nanocarbon allotropes. For detailed information relating to overarching FRET theory and concepts, common applications and recently-developed materials, refer to “FRET—Förster Resonance Energy Transfer” (published in 2013) [27]. Other publications of interest include “Emerging Non-Traditional Förster Resonance Energy Transfer Configurations with Semiconductor Quantum Dots: Investigations and Applications” (published in 2014), which concentrates on the development of FRET with the utilization of quantum dots [28], and “Materials for Fluorescence Resonance Energy Transfer Analysis: Beyond Traditional “Dye to Dye” Combinations” (published in 2006), which gives a critical overview of the major classes of fluorophore materials and their benefits/limitations [29]. Contrary to these literature reviews, all of the examples highlighted in this review focus on unique combinations of carbon-based materials that have not been generally reviewed until now.

## 2. Synthesis and Material Properties

Three types of NCAs that have perhaps had the most significant impact on biosensing/sensing are fullerenes, CNTs, and graphene. Fullerenes, or zero-dimensional (0D) CDs, were first discovered by Richard Smalley and coworkers at Rice University, as well as Kroto and Walton in Sussex, both in 1985 [30], CNTs by Sumio Iijima and coworkers at NEC Corporation’s Fundamental Research Laboratories in 1991 [31] and graphene by Andrew Geim and Kostya Novoselov at Manchester university in 2004 [32,33]. Since their “discovery” or first characterization, numerous NCA fabrication techniques have been developed primarily to improve the scalable nature of the fabrication and, in the case of biosensors, to increase their functionality for interfacing with biorecognition agents.

### 2.1. Carbon Dots

CDs or fullerenes have been synthesized in a variety of shapes *via* numerous fabrication methods. Although fullerenes have a molecular structure of C_60_ (called a “buckyball”), it is very common to produce different masses and isomers, such as C_70_ (it is possible to form C_n_ with *n* > 20, with the most common isomers, C_60_ and C_70_, resembling a soccer ball and a rugby ball, respectively), in a reaction chamber. Consequently, spherical, cylindrical and ellipsoidal fullerenes were synthesized after the discovery of the buckminsterfullerene by Kroto, Curl, Heath, O’Brien and Smalley while they were seeking to explore and understand unidentified interstellar matter by producing carbon plasma [30]. The original method for fullerene synthesis used vaporization of graphite by a high density focused pulse laser with an input energy of approximately 30 mJ [30]. Soon after that, methods, such as evaporating graphite in ~100 Torr of helium atmosphere [34], resistive heating of graphite [35] and catalytic decomposition of acetylene over iron particles and carbon itself at high temperatures of ~800–1000 °C [36,37], were invented for CD growth. Other experimental approaches have been reported to fabricate carbon nanoparticles, such as the carbon arc technique, microwave-plasma chemical vapor deposition, supersonic cluster beam deposition and pulsed laser deposition, among others [38]. As in other cases of growing carbon nanomaterials, growth parameters, such as gas flow, catalyst specification and size and the temperature of growth kinetics, all play a major role in the synthesis process. Coalescing and formation of the carbon rings during the dynamic growth process have also been reported and are currently a subject of much research in the community [39]. While a C_60_ molecule (buckyball) typically has a 0.4-nm inner diameter, 0.7-nm outer diameter and 1-nm π-electron cloud outer diameter, typically, in a reactor, it constitutes 70%, with another ~15% of C_70_ and the remaining 15% constituting all other isomers. Note that the difference in the inner and outer diameter of a C_60_ roughly equals the thickness of graphene, a single sheet of carbon. While C_60_ has 20 hexagons and 12 pentagons, a C_70_ molecule contains 25 hexagons and 12 pentagons. Both C_60_ and C_70_ are *n*-type semiconductors with ~1.6 eV and 1.77 eV bandgap energies, respectively. C_60_ is commonly used as an electron acceptor in layered organic photovoltaics due to its high electron affinity. Using the most sophisticated techniques in nanotechnology, such as aberration-corrected high-resolution tunneling electron microscopy (HRTEM), it is possible to manipulate and tailor the diameter of fullerene to form a giant fullerene using metal catalysts, such as tungsten (W), at high temperatures [40]. Such discoveries, even after two decades, are believed to highlight how much more there is to explore about the unique capabilities and properties of NCAs.

CDs are attractive for use in many optical-based sensing applications due to their inherently strong photoluminescence and resistance to photobleaching [41,42]. In fact, CDs are unique among fluorescent nanoparticles, as they have been shown to exhibit constant photoluminescence for several hours [41] and are considered biocompatible [43]. The biocompatibility of CDs is largely attributed to their low toxicity, as other quantum dots contain concentrations of heavy metals, such as cadmium [44]. Photoluminescent CDs have been experimentally used in a variety of applications, including cell imaging [44,45], pH monitoring [46] and light energy conversion [47].

It is important to note that the method of energy transfer to and from quantum dots/CDs is highly dependent on the spectral overlap between the donor and acceptor molecules [48]. Electron transfer (ET) is referred to in the literature as the “default” quenching process in dots, but FRET dominates when sufficient spectral overlap is present [48]. As CDs are the physically smallest carbon allotrope considered here, speculation leads to the conclusion that it is unlikely that ET is the default quenching mechanism in CNTs or graphene.

### 2.2. Carbon Nanotubes

Cylindrical carbon atom microtubules, now known as CNTs, are made up of *sp^2^*-hybridized carbon atoms and boast extraordinarily high length-to-diameter aspect ratios (with typical diameters of 1 nm for a single wall tube to tens of nm for a multiwall CNT), and were first discovered by Iijima and coworkers in 1993 [49]. This same group identified the smallest CNT to date, having a diameter of only 0.4 nm, in the year 2000 [50]. Originally grown using a DC arc discharge evaporation method of carbon in ~100 Torr argon at ambient temperature, the potential applications of CNTs are nearly limitless: nanoelectronics, nanotechnology, biotechnology, sensors, thermal management, mechanical robustness or various forms of energy storage (e.g., as solar cells, super capacitors) [3,51,52,53,54]. The one-dimensional (1D) geometry of CNTs can even enable the probing of single cells with a unique CNT tip that interfaces with biorecognition agents [55].

Single-walled CNTs (SWCNTs) can be either metallic or semiconducting, and in chemical vapor deposition growth processes, typically, the resultant SWCNTs are 2/3 semiconducting and 1/3 metallic [56]. The rolled geometry, or chirality, of the hexagonal C–C bond networks determine the bandgap, 0 eV (metallic) to 2 eV (semiconducting), of the SWCNTs. SWCNTs fluoresce at near-infrared wavelengths and have shown some biocompatibility, where they experience low absorption of blood and tissue. However, there are toxicity concerns, especially upon inhalation, as SWCNTs have the same length scale as cancer-causing fibers, like asbestos [57]. Multiwall CNTs (MWCNTs) are multiple sheets of graphene rolled up into a tube that displays a metallic electronic character. Multiple growth methods for both SWCNTs and MWCNTS have been developed, including pulsed laser ablation, various forms of chemical vapor deposition (such as microwave power chemical vapor deposition (MWCVD) grown at high temperatures ~800–1000 °C) and various chemical synthesis methods [58,59,60,61,62].

Current growth methods of CNTs using any form of a chemical vapor deposition (CVD) process are typically catalyst-assisted with the use of metal nanoparticle seeds for growth initiation. Such CVD methods use catalyst nanoparticles (e.g., iron, nickel, cobalt, platinum, palladium) deposited onto a surface (e.g., silicon, quartz, copper) that is placed in a high-temperature, low-vacuum furnace, with temperatures upwards of approximately 1000 °C. Subsequently, a line of carbon feedstock gas, such as methane or ethylene [11], is pumped into the furnace for a set amount of time in order to initiate and stop CNT growth. For large-scale nanotube-based device fabrication, nanoparticles are often deposited onto a patterned substrate surface via photolithography or another lithographic process to assist selective area carbon feedstock cracking and, thus, facilitate the nucleation of nanotubes [63].

Worldwide efforts at leading research corporations, such as IBM, national laboratories and at research universities have led CNT technology from scientific fascination to real-world application [64,65]. Such applications include building prototype carbon-based computing devices [66], transparent conductors and glucose-based electrochemical biosensors for monitoring physiological activities [3]. Various scalable sorting techniques for semiconducting *versus* metallic tubes [67], scalable transfer techniques (from source substrate to virtually any arbitrary substrate), scalable aligning techniques coupled with reduced CVD growth temperature and metal catalyst-free CNT growth [68] have all significantly advanced the applications of CNTs. Industrially-scalable processes, such as ink-jet/aerosol printing, screen printing, contact printing and 3D printing, have even been utilized to enable high-throughput and large area fabrication of NCAs [11,69,70].

### 2.3. Graphene

Finally, graphene, the two-dimensional NCA, was discovered decades after CDs and CNTs [71]. This “late” discovery of graphene in 2004 was due in part to graphene’s elusive invisible nature or high optical transparency [72]. Graphene’s unique band structure enables remarkable material properties that include near ballistic electronic transport, high tensile strength and thermal conductivity, relatively low weight and high flexibility [25,32]. Due to its super-flexible nature, graphene can be rolled, stacked, wrapped or otherwise manipulated to form varying geometries, including tubes, spheres and blocks [71]. Graphene’s most exceptional properties occur when sheets or layers are stacked vertically through weak van der Waals interactions in 3D graphite lattice layers numbering less than 10 [73].

Graphene traditionally acts as an energy acceptor in energy transfer, because of its peculiar electronic properties for a two-dimensional material. Its average length of electron-phonon scattering is greater than 2 mm, which is surprisingly long. Consequently, at room temperature, the electron mobility in graphene can exceed 200,000 cm^2^/V/s [74]. Photophysical calculations confirm that energy can be transferred from dyes to graphene without much difficulty, an attribute that makes graphene an excellent quencher of electronically-excited states of dyes [75]. Additionally, theoretical calculations suggest that graphene quenching may be observable at a distance of up to 30 nanometers, thus indicating that graphene is potentially a super quencher with long-range nanoscale energy transfer properties [75].

During the last decade, researchers have focused efforts on the large-scale growth of graphene. They have also developed new methods for isolating single layers of graphene that are much more efficient than the original “scotch tape method” or simple mechanical exfoliation from graphite as devised by Geim and Novoselov [32]. While CVD growth using copper as a catalyst metal source (and a few other metals, such as nickel) is quite common, other potential growth methods, including epitaxial graphene growth on SiC crystals, have demonstrated graphene’s numerous applications [76]. At present, issues such as low temperature-large area graphene growth, catalytic-free graphene growth on arbitrary substrates and large-scale single crystal graphene growth are still active areas of research [77,78,79]. Recently, a low-temperature-modified CVD growth method of graphene at 300–400 °C was demonstrated by Iijima and coworkers that could have significant industrial impact on the large-scale fabrication of functional devices [80]. Despite these advancements, large-scale growth of graphene is still very much an evolving research field.

## 3. Detection of Chemical Compounds

FRET-based sensing has been employed in a wide variety of applications, including those related to the detection of chemical compounds. The concomitance of high quenching efficiency and the biocompatibility of NCA FRET-based sensors permits enhanced sensitivity and low detection limits of chemical compounds. This section demonstrates how chemicals, such as pharmaceuticals, toxic compounds and carcinogens, can be detected with NCA FRET-based biosensors.

**Figure 1:**
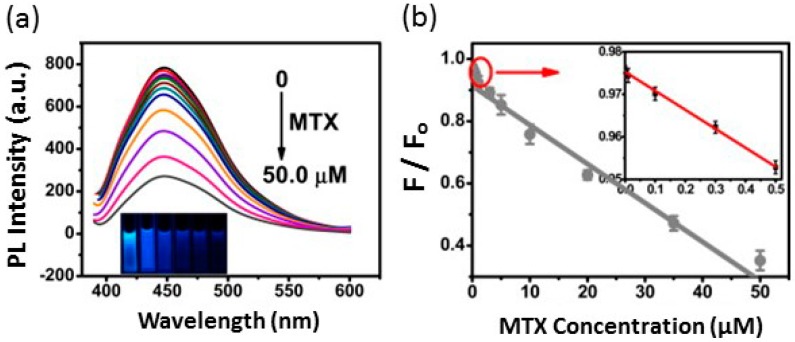
(**a**) The fluorescence spectra of NS-co-doped fluorescent carbon nanodots (NSCDs) as treated with different concentrations of methotrexate (MTX) ranging from 0–50.0 µM; the intensity decreases as the concentration of MTX increases and FRET is inhibited. The inset shows photographs that correspond to the increasing concentrations of MTX; (**b**) The linear relationship between fluorescence and MTX concentration. Reproduced with permission from Wang *et al.* [42]. Copyright 2015 Biosensors and Bioelectronics, Elsevier. PL, photoluminescence.

Pharmaceutical drug testing has been successfully carried out via NCA FRET-based sensors. For example, Wang *et al.* utilized FRET in the development of a biosensor designed to monitor levels of methotrexate (MTX), an anticancer drug, in patients undergoing clinical treatment. Nitrogen and sulfur co-doped fluorescent carbon nanodots (NSCDs) were developed through a green thermal treatment of ammonium persulfate, glucose and ethylenediamine [42]. The prepared dots exhibited a bright blue emission and a high quantum yield of 21.6%, as well as good water solubility, excellent chemical stability and uniform morphology [42]. In this sensor, the NSCDs were quenched via FRET from MTX, and the hydrogen bonds between NSCDs and MTX played a critical role in the quenching effects (see Figure 1). Furthermore, NSCDs have an absorption peak centered at 343 nm and exhibit the excitation spectrum at 382 nm; however, fluorescence spectra can be positively shifted by adjusting an excitation wavelength from 382 nm to 430 nm, which, in turn, greatly decreases the resultant photoluminescence (PL) intensity [42]. Through theoretical calculations, the theoretical distance between donor and acceptor when FRET efficiency is 50% was calculated to be 1.13 nm, while the actual donor-to-acceptor distance was measured as 2.78 nm. The sensor demonstrated high sensitivity and selectivity, a wide linear sensing range of 50.0 µm and a low detection limit of 0.33 nM [42]. Interference testing with the presence of a series of drugs and human blood further proved the accuracy and stability of this FRET-based biosensor. Additionally, due to large fluorescence lifetimes (8.1 ± 0.2 ns), these as-prepared NSCDs could potentially be used in lifetime-based sensing or imaging [42].

Toxic compounds have also been detected via NCA FRET sensors. Yu *et al.* developed a ratiometric fluorescent sensor for the detection of hydrogen sulfide in the body. Ratiometric sensors present strong benefits, as they are self-calibrating and use the ratio between two different fluorophores to detect analytes. In this sensor, CDs served both as the energy donor and also as the anchoring site for the sensing probe, a naphthalimide azide derivative. With the absence of hydrogen sulfide, CDs had an excitation at 340 nm and an emission at 435 nm. When the target was introduced into the solution, the emission peak of 425 nm shifted to an emission band at 526 nm. The shift between donor excitation and acceptor emission spanned 190 nm. Such a large shift in wavelength eliminates the influence of excitation backscattering effects on the fluorescence detection. In the presence of hydrogen sulfide, the probe is reduced and chemically altered from naphthalimide-azide into naphthalimide-amine [46]. This sensor demonstrated strong performance metrics, including a low detection limit of 10 nM and sensing capability across a wide pH range from 4.0 to 9.0 [46].

Finally, a NCA FRET-based sensor for chlortoluron, a widely-used herbicide with known carcinogenic properties that is hazardous to aquatic organisms, was also recently developed. In this sensor, CDs act as energy donors to CdTe energy acceptors. Energy transfer via FRET results in the quenching of CDs, but when chlortoluron is present, the fluorescence of CdTe is quenched in proportion to its concentration. CDs in this sensing platform have maximum absorption and emission peaks at 380 nm and 432 nm, respectively, while CdTe’s are at 495 nm and 570 nm, respectively. Additionally, the quantum yield of CDs was found to be 68%, and that of CdTe was 57%. The sensor demonstrated a linear sensing range of 2.4 × 10^−10^ mol·L^−1^–8.5 × 10^−8^ mol·L^−1^ along with a detection limit of 7.8 × 10^−11^ mol·L^−1^ [81]. 

## 4. Detection of Proteins

As it is important to sense hazardous chemical compounds, it is also vital that proteins can be accurately detected and measured to enhance medical diagnostics and even improve cell imaging techniques. Herein, we discuss uses of FRET to sense proteins, such as thrombin, ferritin and lectin concanavalin A (ConA).

**Figure 2:**
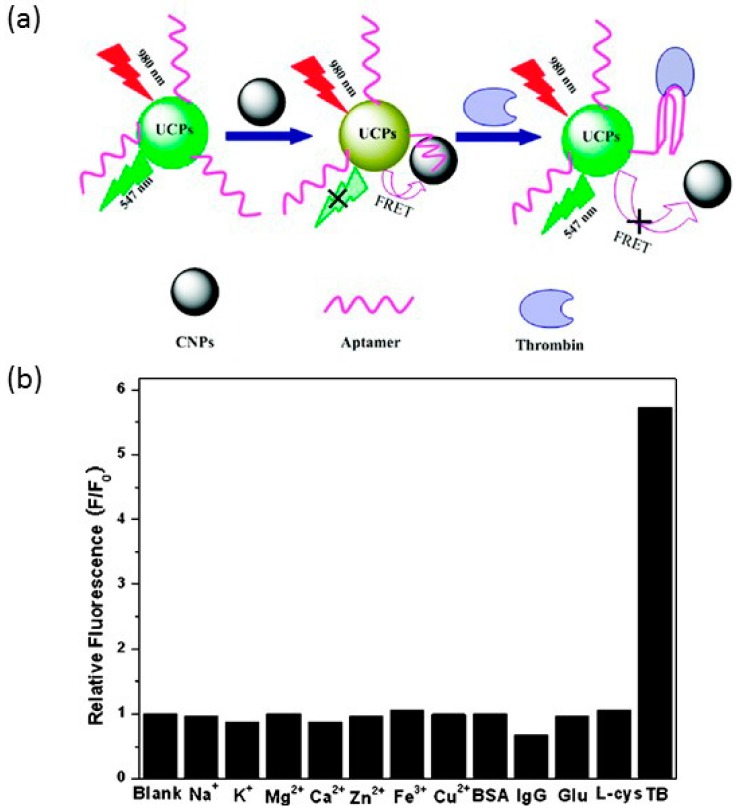
(**a**) Scheme showing the FRET process from upconverting phosphors (UCPs) to CNPs; (**b**) interference testing shows that the fluorescent intensity increases dramatically in the presence of thrombin. Reproduced with permission from Wang, Bao, Liu and Pang [84]. Copyright 2011 American Chemical Society.

Wang *et al.* developed an aptamer biosensor for the detection of thrombin (MW = 28,000 ± 1400 [82]), a blood clotting enzyme [83], in plasma and serum [84]. This sensor was developed on the basis of energy transfer via FRET from upconverting phosphors (UCPs) to carbon nanoparticles [12]. Here, 0.036 mg/mL of CDs were mixed with 0.03 mg/mL UCPs-aptamer in a Tris-HCL buffer. CDs act as energy acceptors, and when thrombin is present in the system, pi-pi interactions are weakened, the upconverting phosphors separate from the CDs and FRET is inhibited (see Figure 2 [43]). This platform was the first application of UCPs and carbon nanoparticles (CNPs) as a donor-acceptor pair, and the developed sensor demonstrated a sensing range of 0.5–20 nM for thrombin with a detection limit of 0.18 nM in an aqueous buffer [84]. Moreover, this sensor acquired a fluorescence quenching rate of 89% under optimized conditions [84].

**Figure 3:**
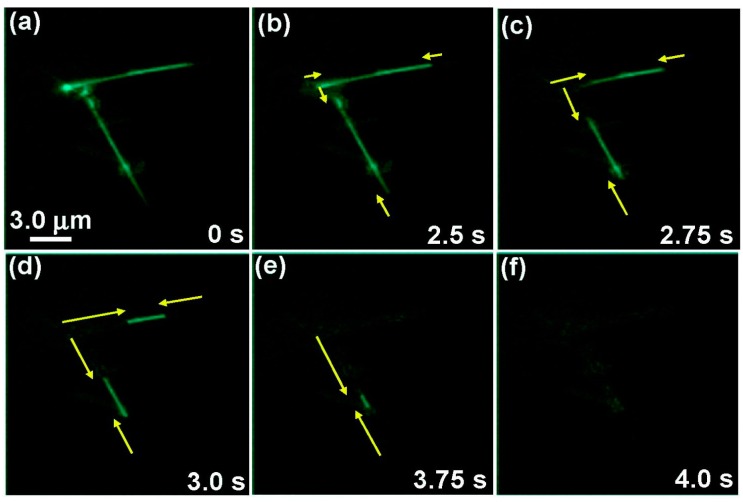
Microscopic images of the NBD nanotubes upon the addition of the fluorescence acceptor dye QSY7, which quenches NBD via FRET. Reprinted with permission from Kameta *et al.* [85]. Copyright 2007 American Chemical Society.

One unique technique that deserves mention involves modifying the inner surfaces of CNTs with fluorescent molecules to monitor the immobilization of ferritin, a spherical protein. This has been used to visualize the dynamic encapsulation and nanofluidic features of ferritin (CALBIOCHEM; 10 mM, 5 mg/mL, MW = 500,000) and DNA in the hollow channel of a modified nanotube and is applicable for optical sensing [85]. FRET was used to visualize three behaviors of guest spherical proteins in nanotube channels through chemical modification, as seen in Figure 3. The fluorescent donor dye, 4-fluoro-7-nitrobenzofurazan (NBD-F), was covalently bonded to an amino group on the inner surface of the nanotube. This interaction triggered fluorescence as NBD-F reacted with amino groups, even though NBD-F had no fluorescence. Using this mechanism, Kameta’s group proved the presence of NBD (from 4-fluoro-7-nitrobenzofurazan) at the inner surface of CNTs [85]. This technique verifies that small molecules can not only be linked to the inner surfaces of CNTs, but they can also be optically visualized instead of CNTs. Such selective binding and visualization are amenable to various drug delivery, medical diagnostics and biosensing applications that utilize CNTs as a protective housing unit for biorecognition agents or drug components [85].

Furthermore, Chen’s group built a maltose-grafted aminopyrene (Mal-Apy) and graphene assembly for the homogeneous detection of the lectin concanavalin A (MW = 255,001, Sigma-Aldrich, St. Louis, MO, USA), a carbohydrate-binding protein [86]. The connection of Mal-Apy and graphene was done through self-assembly by means of pi-stacking interactions between pyrene rings and graphene. Here, the graphene acts through means of FRET to serve as a nanoquencher of the pyrene rings. With the existence of ConA and the competitive binding of ConA with glucose, the recovery of fluorescence was observed due to the destruction of pi-pi interactions between graphene and pyrene rings. The biosensing of ConA in this configuration is selective and follows a linear trajectory over a concentration range of 2.0 × 10^−2^ μM–1 μM with a low detection limit of 0.8 nM [86]. Additionally, the quenching efficiency of Mal-Apy by graphene is surprisingly fast and high, reaching 85% in one minute. Due to these features, this novel sensor is expected to be an excellent platform for protein-carbohydrate studies and has the potential to be utilized in drug screening, biomolecular recognition and disease diagnostics [86].

In another example, water-soluble graphene oxide (GO) was built into a platform for the sensitive and selective detection of proteins and DNA. The strong non-covalent binding of GO with nucleobases and aromatic compounds allows GO to bind dye-labeled ssDNA and fully quench the dye’s fluorescence [87], resulting in 97% quenching of the fluorescence emission. The fluorescence of the selected DNA has an excitation peak at 480 nm and an emission peak at 580 nm. The dye-labeled DNA (5′-AGT CAG TGT GGA AAA TCT CTA GC-FAM-3′ (FAM = fluorescein-based dye)) and the target, thrombin, within a concentration ranging from 5 nm to 10 nm, alters the structure of DNA and its connection with GO, which consequently releases the dye-labeled DNA from GO and recovers the fluorescence [87].

**Figure 4:**
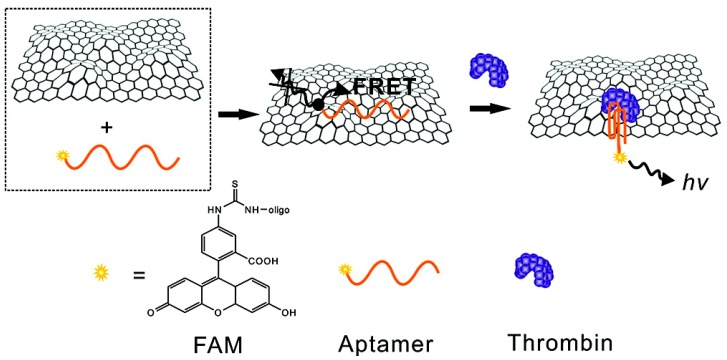
FRET causes the aptamer to bind to graphene, thus quenching the fluorescence of an attached dye. The fluorescence is recovered when quadruplex-thrombin is formed, as it has a weak affinity to graphene, thus removing the dyes from the graphene. Reproduced with permission from Chang *et al.* [88]. Copyright 2010 American Chemical Society.

Through noncovalent assembly between ssDNA and graphene, Chang’s group applied FRET to quench the fluorescence of a dye in yet another example of thrombin detection (Figure 4) [88]. The fluorescence intensity was reported to decrease rapidly with increasing graphene concentration, and the quenching efficiency reached 80% with 0.1 mg/mL of graphene added. When thrombin was added to the system of ssDNA (5′-FAM-GGT TGG TGT GGT TGG-3′) and graphene, recovery of fluorescence occurred. This is attributed to the formation of quadruplex-thrombin, a complex with weak affinity that blocks the binding of dyes to the graphene surface. This mechanism is schematically explained in Figure 4. A low detection limit of 31.3 pM was reported in this research, which was two orders of magnitude lower than those of fluorescent sensors based on CNTs [88].

## 5. Detection of DNA

Carbon nanotubes, carbon dots and graphene have all been utilized to detect DNA through FRET. The detection of single-stranded DNA (ssDNA) and double-stranded (dsDNA) is of great interest in the life sciences, and the following examples demonstrate the variety of approaches that can be taken to accomplish this detection. While the methods and applications of FRET vary in the works presented, the general results show rapid and effective optical sensors for the detection of DNA.

The non-covalent assembly of SWCNTs and dye-labeled single-strand DNA (ssDNA) by pi-stacking between nucleotide bases and SWCNT sidewalls was reported as a new class of fluorescent biosensors, which is able to probe and recognize biomolecular interactions in a homogeneous format [89]. This platform could effectively quench and restore fluorescence when a target is present. It was found in this study that more than 90% of fluorescein derivative FAM’s fluorescence was quenched by nanotubes in the DNA probe concentrations of 50–150 nM. It should be noted that the study also found that fluorescence quenching efficiency decreased considerably as probe concentration increased. To test the feasibility of this method, one 23-base oligonucleotide and a human alpha-thrombin (TMB) binding aptamer were selected for use in this research. The resulting fluorescence emission spectra demonstrated the limit of TMB detection to be 1.8 nM, which is around 10-fold lower than that of the regular dye-quencher pair-labeled aptamers [89]. 

**Figure 5:**
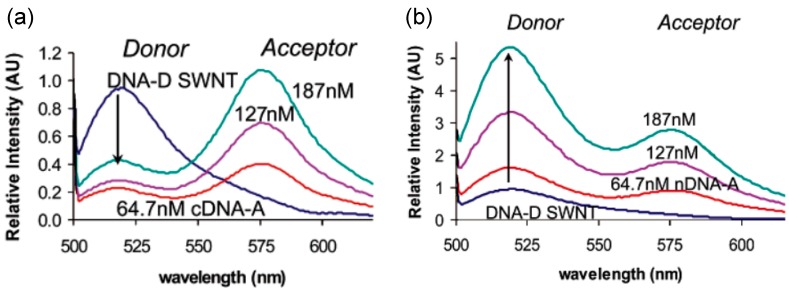
Intensity at donor emission (max 520 nm) of DNA-D-NT. (**a**) Förster resonance energy transfer (FRET) is clearly occurring, as indicated by the two alternating peaks. This, in turn, indicates DNA hybridization on nanotube surfaces. Emission of the donor on DNA-NT decreases with additions of attachment to the acceptor, thus the alternating peaks. The addition of complement conjugated with acceptor (cDNA-A) actually increases the donor emission at higher concentrations; (**b**) No FRET between donor and acceptor appears, indicating that there is no hybridization occurring, and the donor fluorescence remains significantly lower in the presence of cDNA-A than without it. Reproduced with permission from Jeng *et al.* [57]. Copyright 2006, American Chemical Society.

By applying FRET, Jeng’s group confirmed the hybridization of a 24-mer oligonucleotide sequence on the surface of solution-suspended SWNTs, through a SWNT band gap fluorescence modulation made by labeling DNA-SWNT with a fluorescently-tagged complement [57]. This detection is schematically explained in Figure 5. This optical detection method for DNA sequences is selective, direct and, with a detection limit of 6 nM, may have applications in the life sciences and medicine as *in vitro* or *in vivo* detectors of oligonucleotides. This is the first report to optically detect selective hybridization of DNA with its complementary strand directly on the surface of SWNTs [57]. Consequently, this opens possibilities for new types of nanotube-based molecular beacons, sensors, probes and sequencing technologies that do not require analyte labeling [57].

**Figure 6:**
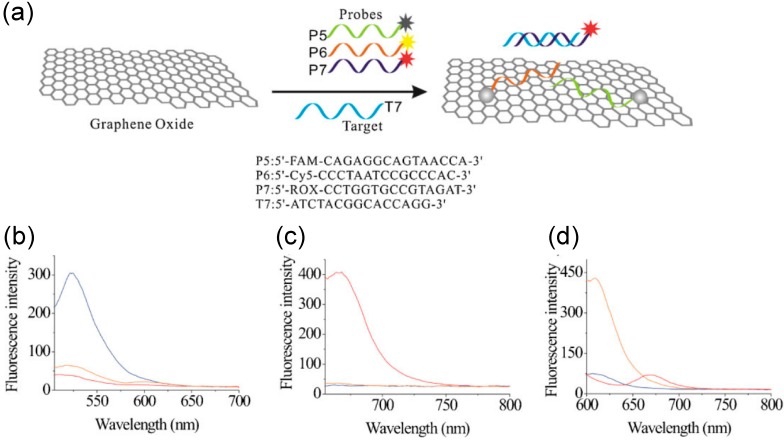
(**a**) Scheme showing DNA analysis using three probes (P5, P6, P7) in the presence of a blue T5 target; (**b**–**d**) fluorescence spectra for multicolor detection showing corresponding wavelengths when the probe is in the presence of different targets: (b) blue T5 at 494/526 nm/nm; (c) red T6 643/666 nm/nm; and (d) orange T7 587/609 nm/nm. Reproduced with permission from He *et al.* [90]. Copyright 2010 Advanced Functional Materials, John Wiley and Sons.

Another novel molecular diagnostic application is the graphene oxide (GO)-based multicolor fluorescent DNA nanoprobe, which is able to detect DNA targets in homogeneous solutions rapidly (within minutes), sensitively and selectively, as seen in Figure 6 [90]. This nanoprobe functions by exploiting interactions between GO and DNA oligonucleotides. In this platform, three probes (P5, P6, P7) are used for three types of tumor-suppressing genes that are exon segments of the p16, p21 and p53 genes. The selection of these three dyes was critical, as they avoid energy transfer among each other [excited at 494, 643 and 587 nm and emitting blue (520 nm), red (670 nm) and orange (608 nm) light, respectively]. When any type of the target was present, the corresponding emission was observed as seen in Figure 6 [90]. All three spectra proved the simultaneous detection of multiple targets in a homogeneous solution, since the emission of each corresponding wavelength was observed while receiving minimal emission from the other two colors [90].

In a final example, a group at Nanjing University utilized FRET from CdTe quantum dots to graphene oxide to identify target DNA [91]. The QDs were capped with mercaptoacetic acid (MPA), and then, a molecular beacon (MB) containing ssDNA was attached, producing MB-QDs with a calculated quantum yield of 8.83% [91]. Once graphene oxide (GO) was introduced, the fluorescence of the MB-QDs reduced to just a tenth of the original value, signifying that GO must be a strong quencher in this case. The team calculated the distance between the MB-QDs and GO before hybridization to be 3.24 nm and determined that the quenching efficiency was 90%. After hybridization with dsDNA, the distance was found to be 9.36 nm and the quenching efficiency 35.3%. The authors concluded that in this work, quenching could be ascribed to FRET at separation distances of less than 7.0 nm, but at distances greater than this, it is due to surface energy transfer [91].

These FRET-based sensors comprised of oligonucleotide linkers between fluorophores or quenches can be modulated according to the length of the connecting linker. As mentioned previously, FRET is typically measureable with donor-to-acceptor length scales of 1–8 nm and typically not measurable above 10 nm. Research by Buckhout-White and coworkers has shown how these FRET length scales correlate with the length (*i.e.*, number of base pairs) of oligonucleotides [92]. In this work acceptor-donor fluorescent pairs (*i.e.*, the cyanine dyes Cy3 (red), Cy3.5 (green), and Cy5 (blue)) were separated by ssDNA consisting of either 9, 18 or 27 bases. These distinctly-sized ssDNA linkers correlated to 0.5 × R_0_, 1.0 × R_0_ and 1.5 × R_0_, respectively, where R_0_ is the Förster distance where 50% energy transfer efficiency is attained. Such distinct ssDNA linker lengths correspond to an estimated FRET efficiency of approximately 95%, 50% and 5% respectively [27,93]. This means that DNA acceptor-to-donor linkers with nine bases or less will completely “turn-on” FRET between attached dyes, while linkers with 27 bases or more will completely “turn-off” FRET. Thus, this research correlating the length of ssDNA linkers and FRET efficiency can be used as an analysis guideline for DNA FRET-based biosensors. For example, the ssDNA capture probes P5, P6 and P7 presented in Figure 6 consist of 15 bases [90]. Thus, when mated with their complimentary fluorescent dye-conjugated ssDNA target probe, it would be expected that FRET between the dye and supporting GO would indeed be “turned-on” with an approximate 50% FRET efficiency. In the MB-QDs presented by Dong and coworkers [91], the DNA linker connecting the dye and quenching GO via a ssDNA hairpin loop opens and closes to turn FRET on and off, respectively. The probe ssDNA linkers were long: the cyclin MB and thrombin aptamer ssDNA hairpin probes contained 33 and 35 base pairs respectively, and, hence, turned FRET off when fully opened. These results are also correlated with the report of Buckhout-White and coworkers, where long ssDNA linkers, *viz.*, 27 linkers or more, turn off FRET between attached donor-acceptor pairs.

## 6. Applications beyond Biosensing

As summarized in the preceding sections, carbon allotropes are attractive for use in sensing capabilities due to their unique material properties. However, resonant energy transfer coupling between fluorophores and carbon allotropes can also be applied to fields other than direct applications in biosensing. In particular, FRET with carbon allotropes has been utilized in cell imaging and light modulation, and it will likely lead to improvements related to light-harvesting applications.

Lee’s group employed a gate-variable optical response in graphene to electrically control resonant energy transfer from colloidal quantum dots to graphene [94]. The device was built with a layer of graphene back-gated with lanthanum fluoride (LaF_3_), a solid-state electrolyte and ionic conductor at room temperature [94]. This film of LaF_3_ formed a thin dipole layer, which, in turn, produced a large capacitance. On top of the graphene was a layer of insulating poly(methyl methacrylate), and the emitter was lead sulfide colloidal quantum dots. This nanoemitter can be built as an extremely small light modulation device and might be potentially used to control light emission beyond the diffraction limit with a superb switching speed [94].

FRET also has been shown to enable maximized energy transfer to certain CNT chiral forms and improve the efficiency of photoluminescence (PL) emissions from these species at low donor concentrations [95]. When presented with excited light at 635 nm, the fluorescent dye Nile blue A fluoresces at an emission maximum of 665 nm. Since SWCNTs with (7,5) chirality have an absorption maximum at 660 nm, it is in resonance with the excited state of Nile blue. Thus it presents a quenching behavior in the presence of (7,5) SWCNT and results in an enhancement in its PL emission, as (7,5) SWCNT has an absorption maximum at 660 nm and is in resonance with the exited state of the dye [95]. Further research also shows SWCNTs with (8,7) chirality absorb at longer wavelengths and present no occurrence of FRET. This result can be potentially used to estimate the concentration of specific chiral forms of nanotubes and provide an impetus for molecular diagnostics and biological applications involving *in vitro* and *in vivo* imaging [96]. In an independent work, the peak energy transfer efficiency in FRET was found to be independent of CNT chirality, which could lead to advancements in light-harvesting applications [96].

## 7. Conclusions

Förster resonant energy transfer with carbon allotropes has extensive potential applications in the medical field ranging from cell imaging to biosensing. This review showcased three prominent NCAs and the ways that they can act as energy donors or acceptors in biosensing systems. The coexistence of unique physiochemical properties (e.g., high photoluminescence and quenching ability, low toxicity) and nanoscale size/dimensions (*viz.* 0D, 1D and 3D) of NCAs are advantageous for a wide variety of FRET-based sensing/biosensing applications, including pH, herbicide, protein and DNA detection, as well as cellular imaging. Furthermore, recent advances, such as enhanced optical detection [57], heightened selectivity [42] and the development of ratiometric sensors, which self-calibrate through the use of two emission bands [46], should make FRET-based detection with NCAs even more enticing to researchers in the future.

Though NCA FRET-based biosensors have been applied in a wide variety of applications, it is important to consider the pros and cons of FRET sensing in general to realize their full potential. FRET-based biosensors are robust and are used in a wide variety of bioanalysis techniques, including molecular beacons [97,98,99], immunoassays [100,101], biomolecular logic [20,21] and active cellular sensing [102,103]. These sensors have been reliably used in living cells and biological serums due in part to their small size (capable of diffusing through cellular membranes) and the inherent high spatial resolution of FRET, *viz.* the ability to monitor nanometer length scales. For example, genetically-encoded FRET biosensors can be used to visualize cellular signaling events in living cells, including protein phosphorylation, G protein activation and BCR-ABL kinase, as well as steady-state glucose monitoring in mammalian cytosol, nuclei and endoplasmic reticulum [104,105,106]. In another example, six-color, time-resolved FRET biosensors have been shown to detect five different tumor biomarkers in a single human serum sample [107,108]. However, FRET signals are usually small and accordingly require careful interpretation and multiple control experiments to ensure accurate sensing [109]. Of course, one of the main limiting factors to FRET is the inability of the energy transfer to occur with lengths approximately greater than 10 nm. Furthermore, donor and acceptor dyes might be of different brightness, which could saturate the image one fluorophore while the other fluorophore is undetectable by optical imaging equipment [109,110]. Furthermore donor-to-acceptor stoichiometry, typically outside the range of 10:1–1:10, could lead to increases of unintended FRET or crosstalk, which would skew the resultant photoluminescence of the acceptors and donors [111]. Acceptor photobleaching, or donor dequenching, can also limit the repeatability of experimental imaging; however, nanoparticles that are resistant to photobleaching, such as QDs, tend to alleviate such concerns [112,113]. Thus, future NCA FRET-based biosensors should have broad potential in bioanalytical biosensing, as well, but certain limitations with FRET sensing in general should be considered during the NCA FRET biosensor design process.

There are several emerging areas of high interest related to NCA FRET-based sensing and biosensing. For example, the juxtaposition of FRET and microfluidics has demonstrated promise in the development of more robust, accurate and sensitive biosensors [114]. Another avenue of anticipated future research is the development of carbon-based FRET sensors that are capable of multiplexing, or detecting multiple events at once [115]. Additionally, future research opportunities also include strategies that permit 3D FRET imaging, which could revolutionize cellular imaging [115]. Of course, the large-scale implementation of such technologies is limited by current NCA fabrication methodologies. Therefore, advances in low-cost, scalable manufacturing of NCAs will likewise help propel the field of FRET sensors/biosensors. Though challenges in this field remain, work related to both FRET and NCAs should lead to a myriad of advances in the field of biotechnology in the foreseeable future. 
